# Jackeline Alger: building capacity in the face of disruption

**DOI:** 10.2471/BLT.23.030723

**Published:** 2023-07-01

## Abstract

Jackeline Alger talks to Gary Humphreys about building public health research capacity in Honduras against a backdrop of conflict and political upheaval.

*Q: The Honduras Medical College (Colegio Médico de Honduras) made you Honduras Physician of the Year in 2019, but you wear many hats. How would you define yourself?*

A: Doctor, researcher, mentor, and perhaps above all capacity builder, since I seem to spend most of my time working to build clinical, technical and research capacity in my country. And when I’m not doing that, I’m thinking about it. However, I certainly started out as a physician, having been inspired by my elder sister, Mucia, the first of many strong women to have had an influence on my life. Like her, I had to leave home to study for my medical degree, which I completed at UNAH (*Universidad Nacional Autónoma de Honduras* (National Autonomous University of Honduras)) in the capital, Tegucigalpa.

*Q: When did you start to take a particular interest in parasitology?*

A: I developed a taste for lab work at UNAH as a research assistant in my spare time, working on medicinal plants for Dr Pablo Cambar, but I later worked on intestinal protozoan parasites under Professor Rina Kaminsky. That work opened up a whole new world to me, not just in the barrel of the microscope but out in the field, where I encountered the health challenges faced by much of the Honduran population.

*Q: Do you mean in terms of exposure to diseases?*

A: Both in terms of exposure to diseases, which range from Chagas disease to dengue, but also in terms of accessing health care. I came to understand just how isolated some communities were. For example, I remember travelling on horseback in the south-east of the country because there were no roads. On one occasion we had to leave the horses on one side of a swollen river during the rainy season. Local people had to pull me across this torrent in a kind of hammock and then put me on a horse on the other side. It made quite an impression on a young woman who had grown up in the city! I went back into similarly remote and isolated settings in my last year as a medical student, which at that time involved a 12-month obligatory placement in an underserved community. I worked on vaccination initiatives close to the border with El Salvador, where a civil war had been going on since 1979.

“[Mentoring] is an opportunity to give back, but also to connect and learn.”

*Q: Did you see anything of the war?*

A: I heard airplanes and shooting from time to time, and I saw the migrants flowing into Honduras. So they were challenging circumstances, but in the middle of it all, there were these committed professionals, doing the best they could with limited resources. It was truly inspiring to see them, and confirmed me in my desire to help them serve the communities for which they had responsibility.

*Q: You in fact did your post-graduate work in the United States. Was there a reason for that?*

A: I was fortunate enough to get a Fulbright Scholarship to study in the United States and also received a TDR (Special Programme for Research and Training in Tropical Diseases) training grant to do research on *Plasmodium vivax*. I felt that it was the best way to acquire the knowledge and skills I needed to help my own country, but I always knew I was coming back.

*Q: You did your doctoral research in Colombia. Why not go straight back to Honduras?*

A: Because there was a lack of infrastructure and regulatory and institutional support there. I did the field work for my doctorate on *Plasmodium vivax* (the parasite that causes malaria) and was concerned that I wouldn’t be able to meet the research objectives I’d set myself. I did work on malaria in Honduras later on, but initially went to the International Center for Medical Research and Training (*Centro Internacional de Entrenamiento e Investigaciones* *Médicas* (CIDEIM)) in Cali, Columbia. CIDEIM was a real powerhouse, with a lot of research going on, but it was also very outward-looking.

*Q: In what sense?*

A: For example, it was working to boost research capacity in the Region of the Americas and was very connected with the international research community. It had – and still has – strong ties with both Tulane and TDR. That desire to connect made a very big impression on me. I also had the great good fortune to work under Donald J. Krogstad and Nancy Saravia, another of those strong women I mentioned.

*Q: How did your experiences feed into your capacity-building work in Honduras?*

A: When I went back to Honduras, I took up a position as a parasitologist at Hospital Escuela, working on a range of projects including work on improving the quality of diagnosis in parasitic diseases. For example, I was part of a multi-centre group led by Professor Pierre Buekens at Tulane University, researching perinatal outcomes of Chagas disease, which showed that rapid tests on umbilical cord blood can be used for international *Trypanosoma cruzi* (the parasite that transmits Chagas disease) seroprevalence studies. At the same time, I was seeing patients and assisting clinical staff in laboratory diagnosis and management of patients with parasitic infections. However, partly because of my experiences in Colombia, I also started teaching on a voluntary basis in clinical postgraduate programmes in UNAH and, in 2008, joined the Scientific Research Unit of the Faculty of Medical Sciences (*Unidad de Investigación Científica de la Facultad de Ciencias Médicas*), where I trained undergraduate and postgraduate students as well as faculty members and health personnel. I also started working with CIDEIM as part of the TDR Regional Training Centre Network for Latin America and the Caribbean. Unfortunately, all that work came to a halt in 2009 following the coup d'état in Honduras.

*Q: More turmoil. How did that affect you personally and professionally?*

A: Well, for all of us it was a shock. I remember it very well – the departure of the president, his attempts to come back, the military blocking the runways with their cars so that his plane could not land. I remember protests in the street, tear gas… Surprisingly perhaps, the work went on and our salaries were paid, but we lost our international partners. In other words, we lost exactly what we needed to maintain our capacity-building momentum. So, it was a very challenging time for me and my family, and the international component didn’t really recover until 2013. We have since been able to take our place in the network and took a leading role in Central America, training professionals from Colombia, Costa Rica, Cuba, El Salvador and Guatemala. I have also trained researchers in Surinam and Tunisia.

“One Health demands that we come out of our silos and connect.”

*Q: You often talk about your mentors and are now a mentor yourself. How important has that been in your career?*

A: In Rina and Nancy, I have been lucky to have two strong, female mentors to guide me in many matters, but being a mentor myself has also been extraordinary. It’s an opportunity to give back, but also to connect and learn. I feel as though I learn as much as I teach by coming into contact with so many different people, and developing the networks and collaborations needed to help move my corner of the health system forward.

*Q: You clearly value networks highly. Can you cite some in which you are active?*

A: I already mentioned my ties with the networks linked with CIDEIM and Tulane, but I would also include the Social Innovation in Health Initiative, also lead by CIDEIM; the Central American Initiative on Snakes (*Iniciativa Centroamericana sobre Serpientes*); the Global Health Network of the University of Oxford; and Healthcare Information for All (HIFA) which is part of the Research4Life initiative. I was voted HIFA Country Representative of the Year in 2015 and 2018, which was a great honour.

*Q: What role has digital technology played in the development of such networks in Honduras?*

A: It has acted as a tremendous facilitator and accelerator, not least in the development of digital platforms such as the Virtual Health Libraries (VHL) which was set up in response to the development of the Internet. Another good example is BIREME (*Biblioteca Regional de Medicina* (Regional Library of Medicine)), which serves as a regional platform for information and knowledge management in the field of health sciences. It was established in 1967, so predates the digital revolution, but it has fully embraced digital technology with various databases, including the Latin American and Caribbean Health Sciences Literature (LILACS) database, one of the most comprehensive sources of scientific and technical health information in the region. BIREME also plays a key capacity-building role by offering training programmes and workshops to strengthen information management skills among health professionals, librarians, and researchers. All of the other networks I mentioned also rely heavily on digital technology. For example, the Global Health Network is a collaborative platform with an online community dedicated to improving global health research and practice, with a particular focus on research capacity-strengthening in low-resource settings.

*Q: If information technology is an accelerator of collaboration what, if anything, acts as a brake in Honduras?*

A: From my perspective, and with specific regard to infectious disease research, institutional fragmentation continues to be a major concern. For example, the three institutions I work closely with – the Honduras Society for Infectious Diseases (*Sociedad Hondureña de Enfermedades Infecciosas*), the Antonio Vidal Institute of Infectious Diseases and Parasitology and the Association for Honduran Parasitology (*Asociación Hondureña de Parasitología*) – all work on infectious and parasitic diseases, but were established separately with different funding mechanisms and serve different groups. We need one integrated organization to meet the challenges we face. More broadly speaking, I would say our siloed approach to addressing public health challenges is a problem. We need to embrace the One Health approach, acknowledging that just about every public health challenge has multisectoral components. We need to come together on issues such as environmental degradation and destruction, which is only accelerating here, even as environmental activists are being killed. One Health demands that we come out of our silos and connect. And we need to do it quickly, because we are running out of time.

