# Corrigendum

**DOI:** 10.2471/BLT.23.100823

**Published:** 2023-08-01

In: Jackeline Alger: building capacity in the face of disruption. Bull World Health Organ. 2023 July 1;101(7):443–444, 

On page 444, the answer to the fifth question should read: “I already mentioned my ties with the networks linked with CIDEIM and Tulane, but I would also include the Social Innovation in Health Initiative, also lead by CIDEIM; the Central American Initiative on Snakes (Iniciativa Centroamericana sobre Serpientes); the Global Health Network of the University of Oxford; and Healthcare Information for All (HIFA). I was voted HIFA Country Representative of the Year in 2015 and 2018, which was a great honour.”

